# Prevalence of Cobalturia Among Adults With Joint Replacements

**DOI:** 10.1001/jamanetworkopen.2021.21758

**Published:** 2021-08-19

**Authors:** Stephen S. Tower, Christina S. Cho, Robert L. Bridges, Bradford D. Gessner

**Affiliations:** 1 University of Alaska, Anchorage; 2 WWAMI (Washington, Wyoming, Alaska, Montana, and Idaho) program, University of Washington School of Medicine, Anchorage, Alaska; 3 Tower Orthopedic and Joint Replacement Clinic, Anchorage, Alaska; 4 Aegis Imaging Consultants, Girdwood, Alaska; 5 EpiVac Consulting Services, Anchorage, Alaska

## Abstract

This cohort study examines the association of cobalt-chrome arthroprosthetic components with adverse reactions to metallic debris, such as cobaltism.

## Introduction

Twenty million North Americans have cobalt-chrome arthroprosthetic components, and 1 million have metal-on-metal hip replacements.^1^ Cobalt is a mitochondrial toxin—encephalopathy and cardiomyopathy (cobaltism) may occur from iatrogenic, industrial, dietary, or arthroprosthetic cobalt exposure.^2,3^ In unexposed populations, the 95th percentile of cobalt levels in urine and blood are 1 part per billion (ppb) and 0.4 ppb, respectively.^3^

Wear and corrosion of cobalt-chrome joint implantations can result in periprosthetic tissue inflammation or necrosis, also known clinically as adverse reactions to metallic debris.^1,4^ Periprosthetic cobalt-chrome metallosis is disseminated systemically and may result in arthroprosthetic cobaltism.^1,3,4^ Systemic cobalt dissemination can result in brain hypometabolism and atrophy; patients with levels of cobalt in blood as low as 1.1 ppb and in urine as low as 4.1 ppb are reported as having cobalt encephalopathy.^5,6^

## Methods

This cohort study analyzed redacted data from 1 orthopedic clinic. The institutional review board at the University of Alaska Anchorage designated the study to be exempt, and patients provided written informed consent for this study. Patients with a suspected hip, knee, or shoulder replacement containing cobalt-chrome were screened with a spot screening of cobalt in urine. Levels of cobalt in blood were then determined for patients with cobalt levels in urine 1 ppb or higher. Patients were designated as cobalt-positive if cobalt levels in urine were 1 ppb or higher or if levels in blood were 0.4 ppb or higher. The reference laboratory test results threshold for reporting cobalt in urine and blood are generally 1 ppb and 0.5 ppb, respectively. Patients with cobalt levels below the reporting threshold in either were assigned half the threshold value. All *P* values were from 2-sided tests and results were deemed statistically significant at *P* < .05. Analysis was conducted using Prism version 9.1.1 (GraphPad). This study followed the Strengthening the Reporting of Observational Studies in Epidemiology (STROBE) reporting guideline.

## Results

A total of 241 patients were screened (mean [SD] age at time of cobalt determination, 68.1 [9.3] years; median duration of exposure to any cobalt-chrome implantation, 11.6 years [range, 2.0-33.2 years]; 117 [48.5%] women); 138 (57%) tested cobalt-positive (mean cobalt level in urine, 12.6 ppb; median, 1.2 ppb). Eleven subjects had no cobalt-chrome implantation (because zirconia and cobalt-chrome femoral-heads are indistinguishable radiographically), none of whom were cobalt-positive. Paired cobalt levels in urine and blood (144 patients) correlated significantly (cobalt in blood = 0.25 × cobalt in urine; *P* < .001). Paired joint-fluid and urine cobalt (57 patients) correlated significantly (joint fluid = 20 × cobalt in urine; *P* < .001). Patients were classified by type, location, number, and brand of their implantation (Table). These classes were risk-grouped based on mean cobalt levels in urine (ie, extreme, greater than 20 ppb; high, between 1 and 21 ppb; low, less than 1 ppb).

**Table.** Risk of Cobalt-Chrome Implantation by Cobalt Levels in Urine and Blood

Implantation	Patients, No. (%)			Urine cobalt level, ppb		Blood cobalt level, ppb	
	Total	Cobalt-negative (urine)^a^	Cobalt-positive (urine)^a^	Mean (SD)	Median (IQR)	Mean (SD)	Median (IQR)
**Extreme-risk joint replacements**							
Metal-on-metal hip replacements and resurfacings	37	0	37 (100)	54.1 (112.6)	9.7 (3.7-28.5)	14.8 (31.7)	2.8 (0.9-7.3)
Extreme-risk total	37	0	37 (100)	54.1 (112.6)	9.7 (3.7-28.5)	14.8 (31.7)	2.8 (0.9-7.3)
**High-risk joint replacements**							
Hips with modular cobalt-chrome neck or cobalt-chrome dual mobility socket	12	2 (17)	10 (83)	NR	NR	NR	NR
Multiple joint replacements	14	2 (14)	12 (86)	NR	NR	NR	NR
Stryker V40 metal-on-plastic hips and Zimmer metal-on-plastic hips	123	55 (45)	68 (55)	NR	NR	NR	NR
Knees bilateral or revision	11	5 (45)	6 (55)	NR	NR	NR	NR
Shoulders	7	3 (43)	4 (57)	NR	NR	NR	NR
High-risk total	167	67 (40)	100 (60)	6.1 (10.2)	1.2 (0.5-6.1)	1.7 (2.9)	0.4 (0.2-1.7)
**Low-risk joint replacements**							
Unilateral primary knees	10	9 (90)	1 (10)	NR	NR	NR	NR
Zimmer 38 mm metal-on-plastic hips, Stryker and Osteonics C-taper metal-on-plastic hips, and DePuy metal-on-plastic hips	16	16 (100)	0	NR	NR	NR	NR
Ceramic-on-plastic hips^b^	11	11 (100)	0	NR	NR	NR	NR
Low-risk total	37	36 (97)	1 (3)	0.4 (0.2)	0.5 (0.2-0.5)	0.2 (0.1)	0.2 (0.1-0.2)
**All groups**	241	103 (43)	138 (57)	12.6 (47.9)	1.2 (0.5-7.9)	3.5 (13.4)	0.4 (0.2-2.3)

Abbreviation: IQR, interquartile range.

^a^ Threshold for cobalt positivity was 1 ppb or higher of cobalt in urine.

^b^ Ceramic heads containing Zirconia are indistinguishable from cobalt-chrome femoral heads radiographically, these 11 patients had no cobalt-chrome implantation.

Extreme-risk implantations (37 patients; mean cobalt in urine, 54.1 ppb; median, 9.7 ppb) were exclusively metal-on-metal hip prosthetics. High-risk implantations (167 patients; mean cobalt in urine, 6.1 ppb; median, 1.2 ppb) consisted of hip constructs with a cobalt-chrome acetabular part (Dual-Mobility) or a modular cobalt-chrome neck, 2 brands of metal-on-plastic hips prone to head-neck taper corrosion, cobalt-chrome–containing joint replacements at different locations, bilateral or revision knee arthroplasties, and shoulder implantations. Low-risk implantations (36 patients; mean cobalt in urine, 0.4 ppb; median, 0.5 ppb) consisted of ceramic-on-plastic hips (with no cobalt-chrome part), several brands and models of metal-on-plastic hips (with cobalt-chrome femoral head) with head-neck tapers not prone to corrosion, and unilateral primary-knee replacements (with 1 to 2 cobalt-chrome parts, no taper junctions, and metal-on-plastic articulation).

## Discussion

Most patients presenting to our clinic with cobalt-chrome hip, knee, or shoulder implantations were cobalturic (ie, cobalt levels in urine of 1 ppb or above) and had cobalt levels in urine or blood in excess of levels associated with encephalopathy. Patients were rarely aware of type, brand, or materials of their replaced joint(s). However, the radiographic appearances of extreme-risk metal-on-metal hips and high-risk modular-neck hips are distinctive (Figure). Only 2 models of joint replacements have been recalled in the US for cobalt-chrome metallosis complications: 1 extreme-risk hip with a metal-on-metal articulation (Johnson & Johnson) and 1 high-risk hip with a modular cobalt-chrome neck (Stryker). Patients with recalled implantations are likely monitored, and many have required revision surgery for cobalt-chrome metallosis complications. Millions of residents of North America implanted with nonrecalled extreme-risk or high-risk implantations are likely not monitored and are likely experiencing cobalturia. Clinicians might consider obtaining a screening of cobalt levels in urine for their patients with a replaced joint presenting with encephalopathy or cardiomyopathy because cobalt might be a remediable etiology.^1,2,3,4,5,6^

**Figure.**
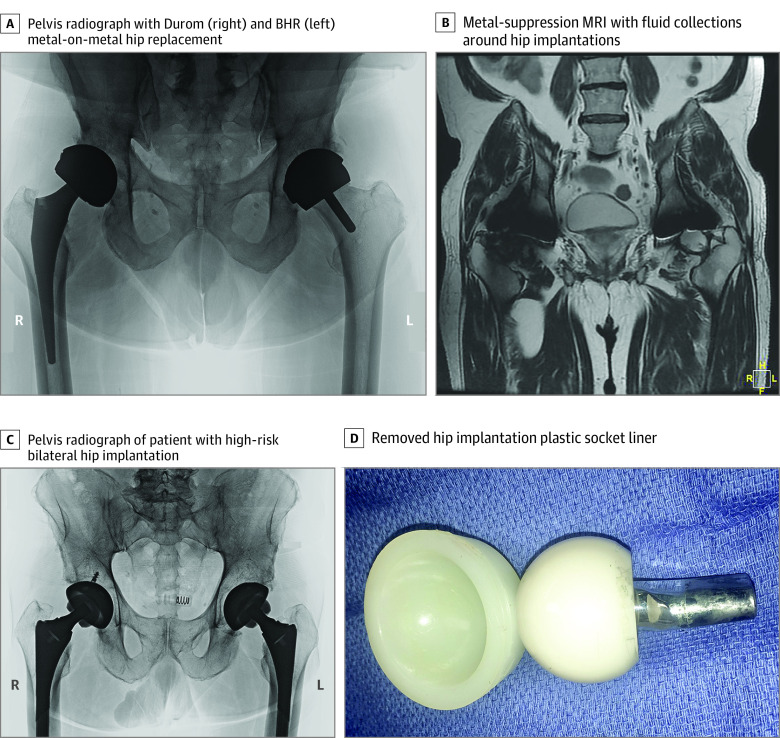
Images of Metal-on-Metal and Modular-Neck Hip Implantations A, The patient with an extreme-risk right metal-on-metal hip replacement implanted in 2007 and a left metal-on-metal hip resurfacing implanted in 2009 presented complaints of tremor, fatigue and forgetfulness, and hip swelling and stiffness. Cobalt levels in urine were 23.5 ppb; in blood, 5.1 ppb; in joint fluid, 440 ppb. Focal brain hypometabolism consistent with toxic encephalopathy was noted on fluorodeoxyglucose-positron emission tomography brain scan. B, Metal-suppression magnetic resonance imaging (MRI) of same patient shows abnormal fluid collections around the medial subtrochanteric area of the right femur and in the joint space of the left hip. C, Patient with high-risk bilateral hips (Microport) with modular cobalt-chrome femoral necks and alumina ceramic femoral heads implanted in 2013, presented with complaints of tremor, fatigue, anxiety, and groin pain. Cobalt levels in urine were 21 ppb; in blood, 4.1 ppb; in joint fluid, greater than 1000 ppb. Focal brain hypometabolism was consistent with toxic encephalopathy noted on fluorodeoxyglucose-positron emission tomography brain scan. D, The explanted left hip plastic socket liner has an alumina ceramic head and a modular cobalt-chrome femoral neck. Note extensive corrosive debris on distal male end of neck.

This study has several limitations. The study was conducted with patients from a single-surgeon clinic. The author-surgeon (S.S.T.) has published the index case report of arthroprosthetic cobaltism from contemporary metal-on-metal hip replacements and is case 1 in that report. The author’s position as patient, surgeon, physician, and researcher introduces the possibility for observer basis. Therefore, it is critical the findings of this report be replicated by other investigators without a personal or financial conflict of interest. Observer basis is unlikely to be a significant factor in this study because reference laboratory determined urine, blood, and joint fluid cobalt concentrations and the type, location, and brand of cobalt-chrome arthroprosthetic implantations studied are not open in interpretation. Multiple coauthors insured the veracity of the data.

## References

[1] Gessner BD, Steck T, Woelber E, Tower SS. A systematic review of systemic cobaltism after wear or corrosion of chrome-cobalt hip implants. J Patient Saf. 2015;15(2):97-104. doi:10.1097/PTS.0000000000000220 26076080 PMC6553976

[2] Smith IC, Carson BL. Trace Elements in the Environment, Volume 6—Cobalt. Technomic Pub; 1981.

[3] Catalani S, Rizzetti MC, Padovani A, Apostoli P. Neurotoxicity of cobalt. Hum Exp Toxicol. 2011;31(5):421-437. doi:10.1177/0960327111414280 21729976

[4] Jones DA, Lucas HK, O’Driscoll M, Price CH, Wibberley B. Cobalt toxicity after McKee hip arthroplasty. J Bone Joint Surg Br. 1975;57(3):289-296. doi:10.1302/0301-620X.57B3.289 1158940

[5] Clark MJ, Prentice JR, Hoggard N, Paley MN, Hadjivassiliou M, Wilkinson JM. Brain structure and function in patients after metal-on-metal hip resurfacing. AJNR Am J Neuroradiol. 2014;35(9):1753-1758. doi:10.3174/ajnr.A3922 24722312 PMC7966284

[6] Bridges RL, Cho CS, Beck MR, Gessner BD, Tower SS. F-18 FDG PET brain imaging in symptomatic arthroprosthetic cobaltism. Eur J Nucl Med Mol Imaging. 2019;47(8):1961-1970. doi:10.1007/s00259-019-04648-2 31863138 PMC7299907

